# Endometrial stromal cell ferroptosis promotes angiogenesis in endometriosis

**DOI:** 10.1038/s41420-022-00821-z

**Published:** 2022-01-17

**Authors:** Guojing Li, Yu Lin, Yili Zhang, Nihao Gu, Bingxin Yang, Shan Shan, Na Liu, Jing Ouyang, Yisai Yang, Feng Sun, Hong Xu

**Affiliations:** 1 International Peace Maternity & Child Health Hospital, Shanghai Municipal Key Clinical Speciality, Institute of Embryo-Fetal Original Adult Disease, School of Medicine, Shanghai Jiao Tong University, Shanghai, 200030 China; 2 Shanghai Key Laboratory of Embryo Original Diseases, Shanghai, 200030 China

**Keywords:** Cell death, Mechanisms of disease, Urogenital reproductive disorders

## Abstract

Endometriosis, a chronic disorder characterised by the presence of endometrial-like tissue outside the uterus, is associated with iron overload and oxidative stress in the lesion. Although it is well established that iron overload can trigger ferroptosis, the results of previous studies on ferroptosis resistance and ferroptosis in endometriotic lesions are paradoxical. Here, we found that some stromal cells of the cyst walls that were in contact with the cyst fluid underwent ferroptosis. Surprisingly, endometrial stromal cell ferroptosis triggered the production of angiogenic, inflammatory and growth cytokines. In particular, angiogenic cytokines, such as vascular endothelial growth factor A (VEGFA) and interleukin 8 (IL8), promoted human umbilical vein endothelial cell (HUVEC) vascular formation in vitro. Moreover, we found that inhibition of p38 mitogen-activated protein kinase/signal transducer and activator of transcription 6 (p38 MAPK/STAT6) signalling represses VEGFA and IL8 expression when endometrial stromal cells undergo ferroptosis. Notably, VEGFA and IL8 showed localised expression and were significantly upregulated in ectopic lesions compared to control and eutopic endometrium samples from patients with endometriosis. Thus, our study reveals that endometrial stromal cell ferroptosis in the ovarian endometrioma may trigger cytokine secretion and promote angiogenesis of adjacent lesions via paracrine actions to drive the development of endometriosis, providing a rationale for translation into clinical practice and developing drugs for endometriosis.

## Introduction

Endometriosis is an oestrogen-dependent disease, characterised by the presence of endometrial glands and stroma outside the uterine cavity. It is a chronic inflammatory disorder affecting approximately 10% of women of reproductive age, with an estimated 200 million affected individuals worldwide [1]. Among patients with endometriosis, about 40–50% have fertility problems and 50% suffer major pelvic pain, affecting the health and quality of the life of patients and causing a major economic burden [2, 3]. Although it has been generally accepted that the development of endometriosis is closely associated with hormones, inflammation, dysfunctional immunity, oxidative stress, genetic and epigenetic factors as well as environmental factors, the pathogenesis of endometriosis has not been completely elucidated [4].

Ferroptosis, a new type of regulated cell death, is triggered by the iron-catalysed process of lipid peroxidation initiated via nonenzymatic (Fenton reactions) and enzymatic mechanisms (lipoxygenases) [5]. It is characterised by small dysmorphic mitochondria with decreased cristae and condensed and ruptured outer membranes and is closely related to iron, polyunsaturated fatty acid, and amino acid metabolism, and glutathione, phospholipid, coenzyme Q10, and NADPH biosynthesis [6, 7]. Ferroptosis is modulated by intracellular iron overload. However, in endometriosis, endometrial cells are not destroyed but instead survive, implant, and grow in the ectopic lesions that contain high levels of iron as a result of repeated bleeding episodes and the gradual accumulation of menstrual debris and antiquated blood in the cyst fluid [8]. Thus, it was hypothesised that endometrial cells can resist iron-mediated ferroptosis [9]. In addition, a recent study reported that ectopic endometrial stromal cells (ESCs) were more sensitive to erastin-induced ferroptosis [10]. Therefore, ferroptosis has drawn immense attention as a promising target for new therapeutic strategies.

Recently, a wide range of angiogenic, inflammatory, and growth-stimulating cytokines have been detected in the serum, peritoneal fluid, and endometrium of patients with endometriosis, suggesting the potential role of inflammation in the progression of this disorder [11]. It has been reported that elevated expressions of vascular endothelial growth factor (VEGF) and interleukin 8 (IL8) induce the production of vascular endothelial cells in ectopic endometrial lesions and that anti-VEGF/VEGF receptor treatments suppress the development of endometriosis in animal models [12, 13]. Interleukin‐1β (IL‐1β) enhances endometriotic cell proliferation, decreases apoptosis, and induces the secretion of IL6 and IL8 in endometriotic tissues, leading to increased proliferation in endometriosis [14, 15]. Ferroptosis induction has been reported to be associated with increased IL6 and IL8 expression [16]. To reduce iron overload, injection of deferoxamine, an iron chelator, into a murine endometriosis model decreased inflammation and limited lesion proliferation [17]. Further work demonstrated that treatment with the ferroptosis inhibitor, N-acetylcysteine (NAC), reduced the volume and weight of endometriotic lesions induced in rodents compared to the controls [18]. In addition, an observational cohort study showed the effectiveness of NAC for endometriosis treatment without side effects [19]. These studies reveal a contrasting ferroptosis mechanism underlying endometriosis, suggesting that ferroptosis may be a double-edged sword in endometriosis. Although it is a promising treatment target, it may also be closely correlated with inflammation and the progression of endometriotic lesions.

The role of ferroptosis in endometriosis has not yet been systematically examined. In this study, we observed ferroptosis in ectopic lesions. We found that ferroptosis of ESCs induced VEGFA and IL8 secretion, and explored its potential effects on angiogenesis of adjacent lesions during the development of endometriosis. Our results provide new insights into ferroptosis in endometriosis, which can be translated into clinical practice.

## Results

### Ferroptosis detection in endometriotic cyst

**Fig. 1:**
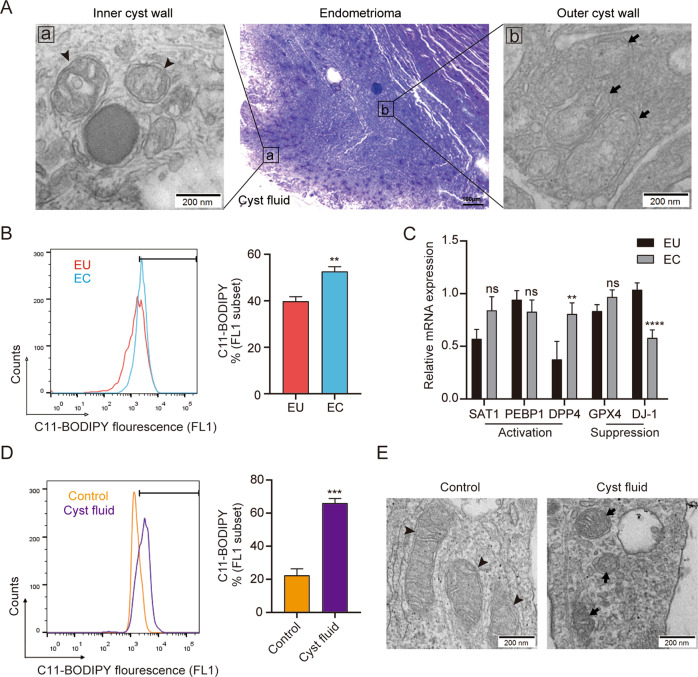
Ferroptosis detection in endometriosis. **A** Representative transmission electron microscopy images of the ultrastructure of the inner and outer walls of mitochondria of endometrioma. **B** Single stromal cells were isolated from the paired eutopic and ectopic endometria and their lipid ROS accumulation was assayed using flow cytometry and C11-BODIPY. Representative data and statistical analyses from five independent experiments are shown. **C** The relative mRNA expression levels of three upregulated (SAT1, PEBP1, and DPP4) and two downregulated (GPX4 and DJ-1) ferroptosis-related genes were compared between the paired eutopic and ectopic endometria (*n* = 24). **D**, **E** ESCs were cocultured with diluted cyst fluid (1:1) for 12 h in vitro. The lipid ROS levels were examined and transmission electron microscopy of mitochondria ultrastructure was analysed. Arrowheads indicate deformed mitochondrial structures, while arrows point to normal mitochondrial structures. Comparisons were made using the two-tailed, Student’s *t* test (**B**–**D**). **P < 0.01, ***P < 0.001, ****P < 0.0001. ns not statistically significant, EU eutopic endometrium from patients with endometriosis, EC ectopic lesions from patients with endometriosis, GPX4 glutathione peroxidase 4, SAT1 spermidine/spermine N1-acetyltransferase 1, PEBP1 phosphatidylethanolamine-binding protein 1, DPP4 dipeptidyl-peptidase-4, ESCs endometrial stromal cells, ROS reactive oxygen species.

Endometriotic cysts exhibit localised iron overload and persistent oxidative stress [20], which may trigger ferroptosis. However, it is not clear whether ferroptosis occurs in endometriotic lesions. As ferroptosis is specifically characterised by mitochondria that appear smaller than normal with increased membrane density, this feature can be used to distinguish ferroptosis from apoptotic or necrotic death, or autophagy [7]. Using transmission electron microscopy (TEM), we could observe shrunken mitochondria with increased membrane density and reduced mitochondrial cristae on the inner surface of the cyst walls (Fig. 1Aa). However, the same mitochondrial changes were not observed on the outer cyst walls (Fig 1Ab). In addition, lipid reactive oxygen species (ROS) accumulation—which plays a central role in the ferroptosis pathway in eukaryotic organisms [21], was measured among eutopic endometrium and lesion cyst cells by flow cytometry using the fluorescent probe C11-BODIPY. The results demonstrated that the endometriotic cyst showed increased lipid peroxidation compared to eutopic endometrium (39.80 ± 1.95% vs. 52.64 ± 2.04%, *P* < 0.01) (Fig. 1B). Moreover, we investigated the expression of important ferroptosis-related genes, such as *SAT1*, *PEBP1* and *DPP4*—which drive ferroptosis, and *GPX4* and *DJ-1*—which suppresses it, upon their activation [22–24]. We found that the level of *DPP4* was higher in ectopic lesions, whereas that of *DJ-1* was lower in lesions than in eutopic endometrium (Fig. 1C). It has been reported that DPP4 controls lipid metabolism, while DJ-1 is associated with glutathione metabolism during ferroptosis [22, 24]. However, no significant differences in GPX4, SAT1 and PEBP1 expression levels were detected between the two groups.

Furthermore, to simulate the microenvironment of ectopic endometrial cells, we co-cultured primary ESCs with diluted cyst fluid (1:1) for 12 h. Surprisingly, we detected an obvious elevation of lipid ROS levels (22.33 ± 4.02% vs. 66.03 ± 2.77%, *P* < 0.001) (Fig. 1D) and observed smaller mitochondria with increased membrane density (Fig. 1E), implying that the contents of endometrioma might trigger ferroptosis in the ectopic endometrium. Altogether, these results suggested that ferroptosis does occur in endometriotic cysts.

### Transcription profiles of ESCs following erastin-induced ferroptosis in vitro

**Fig. 2:**
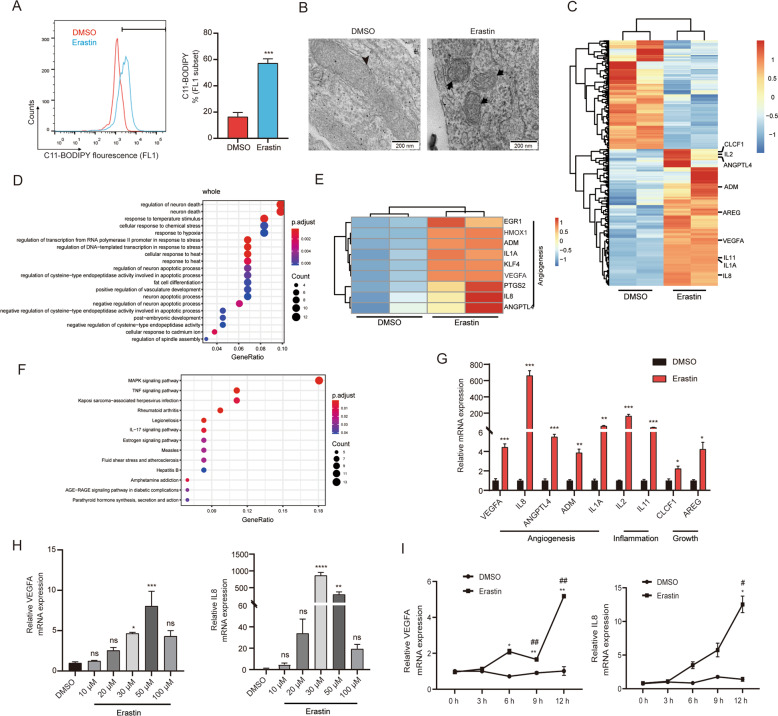
Transcription profiles and verification of erastin-treated ESCs. Primary ESCs were treated with erastin (30 µM) or DMSO for 12 h. **A** Lipid ROS levels were assessed using flow cytometry and C11-BODIPY. Representative data and statistical analyses from three independent experiments are shown. **B** Transmission electron microscopy analysis of mitochondria ultrastructure in ESCs under erastin treatment. **C** Heatmap displaying a subset of DEGs in ESCs treated with 30 µM erastin for 12 h (≥2‐fold, *P* < 0.05). GO (**D**) and KEGG pathway enrichment (**F**) analyses on DEGs in response to erastin-induced ferroptosis. **E** Heatmap of angiogenesis-related DEGs. **G** RT-qPCR analysis was used to validate the DEGs, which included angiogenic cytokines (VEGFA, IL8, ANGPTK4, ADM, and IL1A), inflammatory (IL2 and IL11) and growth factors (CLCF1 and AREG) (*n* = 3, compared with DMSO). **H**, **I** The ESCs were treated with erastin at different concentrations (10, 20, 30, 50, and 100 µM) for 12 h and for different time periods (0, 3, 6, 9, and 12 h) using 30 µM erastin. VEGFA and IL8 mRNA expressions were detected using RT-qPCR and statistical analysis from three independent experiments. * Compared with DMSO, # compared with 0 h. Arrowheads indicate deformed mitochondrial structures, while arrows point to normal mitochondrial structures. Comparisons were made using the two-tailed, Student’s *t* test (**A**, **G**), one-way ANOVA (**H**) and two-way ANOVA (**I**). *P < 0.05, **P < 0.01, ***P < 0.001, ****P < 0.0001, ^#^*P* < 0.05, ^##^*P* < 0.01. ns not statistically significant, ESCs endometrial stromal cells, DMSO dimethyl sulphoxide, ROS reactive oxygen species, GO Gene Ontology, KEGG Kyoto Encyclopaedia of Genes and Genomes, DEGs differentially expressed genes, VEGFA vascular endothelial growth factor A, IL8 interleukin 8, ANGPTK4 angiopoietin-like protein 4, ADM adrenomedullin, IL1A interleukin 1A, CLCF1 cardiotrophin-like cytokine factor 1, AREG amphiregulin.

To explore why endometrial cells of ectopic lesions survive and grow in spite of stromal cell ferroptosis and also if ESC ferroptosis benefits the progression of ectopic lesions, we investigated erastin-induced ESC ferroptosis. First, to explore the susceptibility of ESCs to erastin-induced ferroptosis, we treated primary ESCs with erastin at different concentrations (10, 20, 30, 50, and 100 µM) under a time gradient (0, 3, 6, 9 and 12 h). Obvious ESC morphological changes were observed after treatment with 30 µM erastin for 12 h (Supplementary Fig. 1). We additionally found markedly elevated lipid ROS accumulations (16.47 ± 3.21% vs. 57.20 ± 3.38%, *P* < 0.001) and notable mitochondrial changes in this culture condition (Fig. 2A, B), indicating that erastin caused ferroptotic cell death in ESCs. To confirm the concrete effect of ferroptosis, ESCs were treated with either DMSO or 30 µM erastin, and total RNA derived from these cells was subjected to RNA sequencing. Bioinformatic analysis indicated that following erastin treatment, 352 transcripts were significantly upregulated or downregulated (≥2‐fold, *P* < 0.05) in ESCs, and multiple secretory factors known to be associated with angiogenesis, inflammation, and growth were observed in the list of upregulated entities including VEGFA, IL8, angiopoietin-like protein 4 (ANGPTK4), adrenomedullin (ADM), IL1A, IL2, IL11, cardiotrophin-like cytokine factor 1 (CLCF1), and amphiregulin (AREG) (Fig. 2C, G), consistent with previous studies showing that ferroptosis accelerates inflammation [25]. We performed gene ontology (GO) enrichment analysis of differentially expressed genes (DEGs). In addition to enrichment in neuronal death, stress response, and apoptotic processes, DEGs were also enriched in vasculature development and cell differentiation (Fig. 2D and E). In addition, Kyoto Encyclopaedia of Genes and Genomes (KEGG) pathway enrichment analysis demonstrated that these DEGs were mainly involved in MAPK signalling (Fig. 2F). We verified the upregulation by examining the levels of a few secretory factors, which also showed significantly higher expression after treatment with erastin (Fig. 2G). Thus, we can conclude that erastin-induced ESC ferroptosis in turn induced the secretion of angiogenic, inflammatory, and growth factors, which may be associated with angiogenesis and the progression of ectopic lesions.

### ESC ferroptosis triggers angiogenesis in vitro

**Fig. 3:**
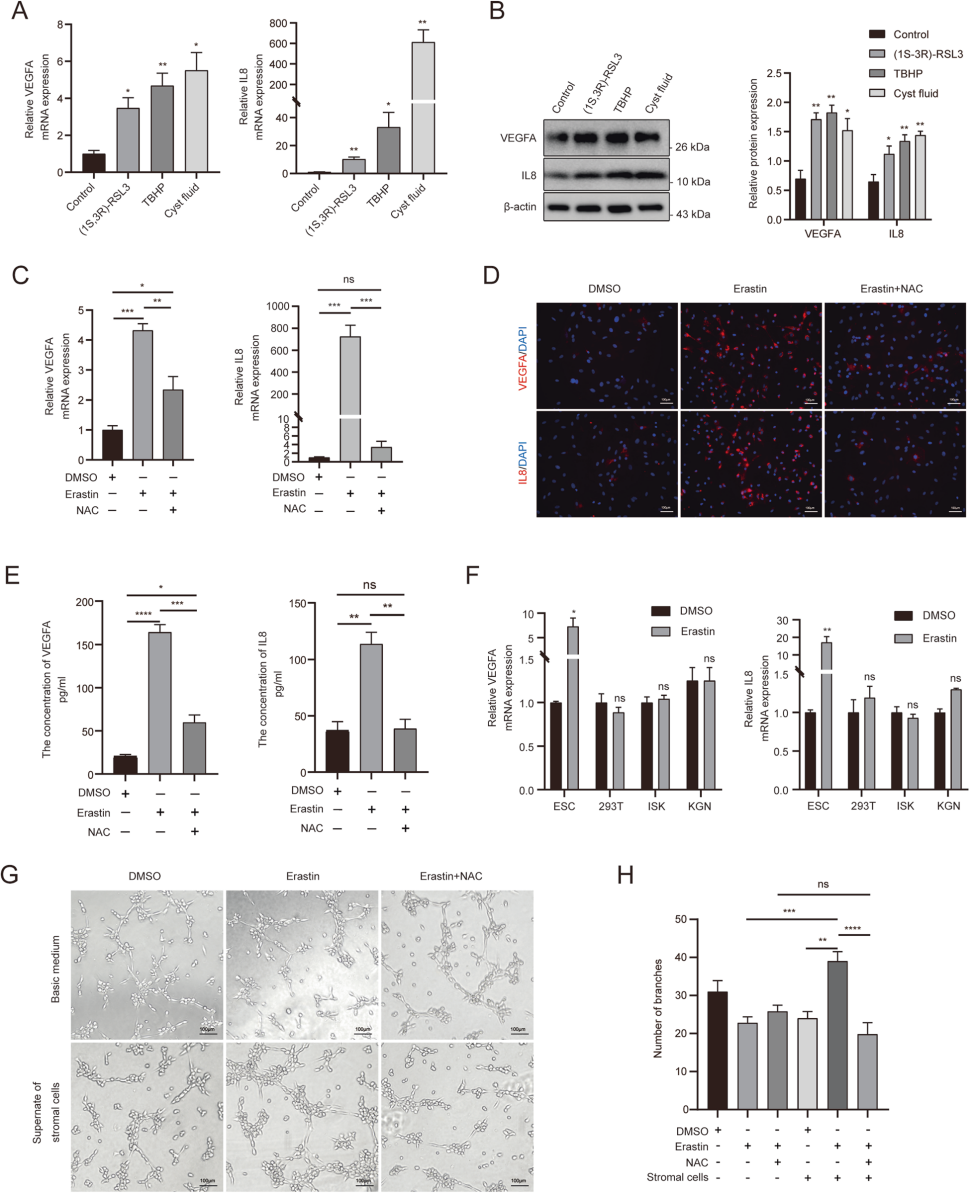
ESC ferroptosis induced VEGFA and IL8 production and promoted angiogenesis. **A**, **B** ESCs were incubated with several ferroptosis inducers, such as (1S,3R)-RSL3 (10 µM), TBHP (20 µM), and the diluted cystic fluid (1:1), for 12 h. VEGFA and IL8 expression levels were detected using RT-qPCR and western blot. Representative data and statistical analysis from three independent experiments are shown. **C**–**E** ESCs were treated with 30 µM erastin in the presence of the bioactive inhibitor NAC (10 µM) for 12 h. VEGFA and IL8 expressions were measured using RT-qPCR, IF, and ELISA. All the statistical analysis was from three independent experiments. **F** Other erastin-treated cell lines (293T, ISK, and KGN) and VEGFA and IL8 mRNA expression levels were detected using RT-qPCR (*n* = 3, compared with DMSO). **G** The angiogenic ability of HUVECs was tested by coculturing with the supernatant from erastin- and NAC-treated ESCs or with a basic medium containing erastin and NAC. Representative micrograph images are shown. **H** The number of branches was analysed (*n* = 5). Comparisons were made using the two-tailed, Student’s *t* test (**F**) and one-way ANOVA (**A**–**C**, **E**, and **H**). *P < 0.05, **P < 0.01, ***P < 0.001, ****P < 0.0001. ns not statistically significant, ESC endometrial stromal cell, DMSO dimethyl sulphoxide, VEGFA vascular endothelial growth factor A, IL8 interleukin 8, NAC N-acetylcysteine, IF immunofluorescence, HEK293T human embryonic kidney cell line, ISK human endometrial cancer cell line Ishikawa, KGN human ovarian granulosa cell tumour cells, HUVECs human umbilical vein endothelial cells, TBHP tert-Butyl hydroperoxide solution.

VEGFA and IL8 are pivotal angiogenic factors [26, 27] playing an essential role in the pathological progression of endometriosis [12]. To confirm that ESC ferroptosis induced angiogenic cytokine expression (VEGFA and IL8), the primary ESCs were first treated with erastin at different concentrations (10, 20, 30, 50 and 100 µM) and for different time periods (0, 3, 6, 9, and 12 h). Compared to the control, the expression of VEGFA and IL8 increased significantly upon erastin treatment from 30 to 50 µM and in a time-dependent manner from 0 to 12 h (Fig. 2H, I). Various ferroptosis inducers, such as (1S,3R)-RSL3, TBHP, and the endometriotic cyst fluid were used to treat ESCs. (1S,3R)-RSL3 is another potential ferroptosis agonist that directly inhibits GPX4, and TBHP was used to simulate oxidative stress conditions, which ultimately resulted in considerable lipid peroxidation [6, 28]. As shown in Fig. 3A, B, the mRNA and protein levels of VEGFA and IL8 were significantly upregulated under the three conditions. In addition, as a precursor of intracellular antioxidant glutathione [29], NAC rescued cell death morphologically (Supplemental Fig. 2A) and inhibited VEGFA and IL8 induction by erastin at the mRNA, intracellular, and secretory protein levels (Fig. 3C–E). These findings suggest that ESC ferroptosis induces VEGFA and IL8 production. Investigations in several cell lines including 293T, ISK, and KGN also revealed that VEGFA and IL8 levels were only upregulated in primary ESCs (Fig. 3F), implying a unique mechanism of ESC ferroptosis. We next examined the effect of stromal VEGFA and IL8 on HUVEC vascular formation using conditioned media derived from erastin-treated ESCs. The Matrigel tube formation assay showed that HUVEC tube-like structure formation (measured based on the total number of branches) was considerably enhanced in cells cultured with the medium from erastin-induced ESCs (erastin-treated ESCs group vs DMSO-treated ESCs group, 39 ± 2.51 vs. 24 ± 1.76, *P* < 0.01; erastin-treated ESC group vs. erastin with basic medium group, 39 ± 2.51 vs. 22.8 ± 1.59, *P* < 0.001) (Fig. 3G, H). However, the angiogenesis-promoting effects of ferroptosis were obviously abrogated in the presence of NAC (NAC- and erastin-treated ESCs group vs. erastin-treated ESCs group, 19.8 ± 3.06 vs. 39 ± 2.51, *P* < 0.0001) (Fig. 3G, H). These data indicate that ESC ferroptosis might promote angiogenesis of surrounding tissues through the release of VEGFA and IL8, thus contributing to primary lesion survival.

### The p38 MAPK/STAT6 pathway is involved in ferroptosis-induced VEGFA and IL8 induction

**Fig. 4:**
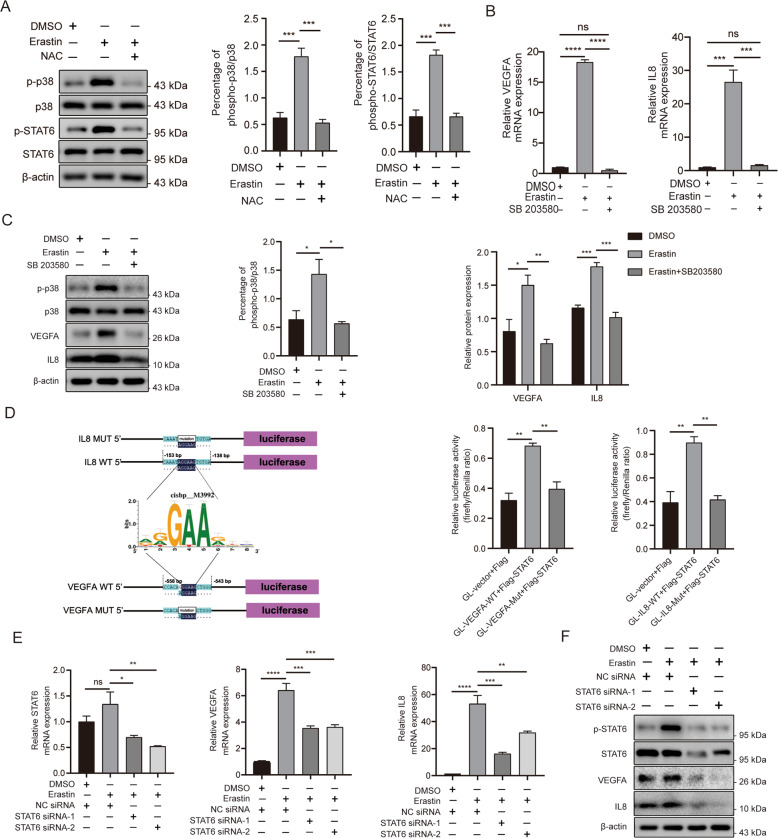
p38 MAPK/STAT6 pathway is involved in ferroptosis-induced VEGFA and IL8 upregulation. **A** ESCs were incubated with erastin and NAC, and the activation of p38 and STAT6 was examined using western blot. **B**, **C** The p38 inhibitor SB203580 (10 µM) was added to erastin-treated ESCs and the activation of p38 was measured using western blot. VEGFA and IL8 expression and secretion were measured using RT-qPCR, western blot (*n* = 3). **D** The prediction model of enriched motifs for DNA-binding activators of STAT6 bind both the TSSs of VEGFA and CXCL8 and is validated using luciferase reporter assays. **E**, **F** ESCs were transfected with STAT6 siRNAs for 48 h and then incubated with erastin for 12 h. The transfection efficiency and expression of VEGFA and IL8 were detected using RT-qPCR and western blot (*n* = 3). Comparisons were made using one-way ANOVA (**A**–**F**). *P < 0.05, **P < 0.01, ***P < 0.001, ****P < 0.0001. ns not statistically significant, p38 MAPK/STAT6 p38 mitogen-activated protein kinase/signal transducer and activator of transcription 6, ESCs endometrial stromal cells, VEGFA vascular endothelial growth factor A, IL8 interleukin 8, NAC N-acetylcysteine, TSSs transcription start sites, CXCL8 C-X-C motif chemokine ligand 8.

We further investigated the mechanism of ferroptosis-induced VEGFA and IL8 upregulation in ESCs. As KEGG pathway enrichment analysis demonstrated that the DEGs of erastin-treated ESCs were mainly involved in the MAPK signalling pathway (Fig. 2F), we first examined the activation of the p38 MAPK signalling pathway using western blot. Upon treatment of ESCs with erastin, the phosphorylation levels of P38 were elevated but attenuated by the addition of NAC (Fig. 4A). We then treated the cells with SB203580, a specific inhibitor of p38 MAPK, and noted that the p38 inhibition significantly decreased erastin‐induced VEGFA and IL8 expression (Fig. 4B, C). Furthermore, analysis of the sequences within 5 kb from the transcription start sites (TSSs) of VEGFA and IL8 using RcisTarget [30] identified enriched motifs for DNA-binding activators (cisbp _ M3992 motif-binding STAT6) both in the TSSs of VEGFA and IL8 (Fig. 4D). To address this, we selected the region 2000 bp upstream of VEGFA and IL8 TSSs or mutated the M3992 motif, fused these sequences to a reporter gene expressing firefly luciferase, and then co-transfected 293T cells with them and the STAT6 expression plasmid. The results showed that STAT6 specifically activated the firefly luciferase of the wild-type promoter, but had no effect on the reporter plasmid (Fig. 4D). Then, we detected STAT6 activation upon erastin and NAC treatment (Fig. 4A), and using siRNA we knocked down STAT6 in erastin-treated ESCs. This partially suppressed VEGFA and IL8 expression, suggesting that STAT6 contributes, at least in part, to ferroptosis-mediated VEGFA and IL8 production (Fig. 4E, F). Collectively, the above results indicate that the p38 MAPK/STAT6 signalling pathway contributes to ferroptosis-mediated VEGFA and IL8 expression.

### Verification of VEGFA and IL8 expression in biospecimens of patients with endometriosis

**Fig. 5:**
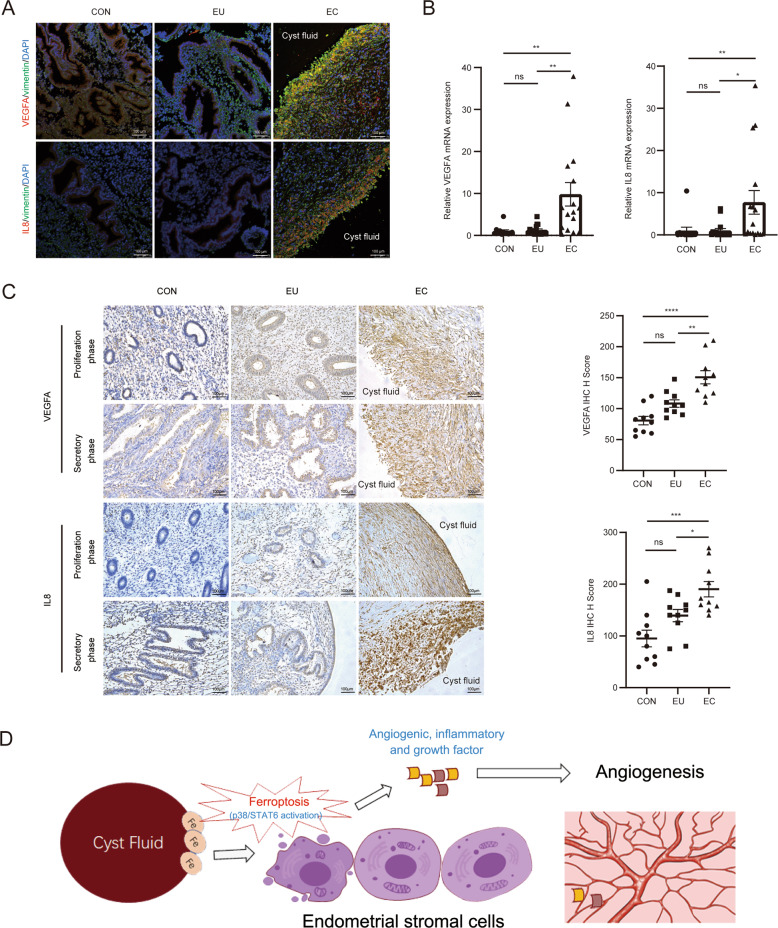
VEGFA and IL8 expression levels in patients with endometriosis. **A** Immunofluorescence images of control, eutopic endometrium, and ectopic lesion samples were co-stained for VEGFA or IL8 (red) and vimentin (green). Nuclear DNA was counterstained with DAPI (blue). **B**, **C** VEGFA and IL8 expression levels of control, eutopic and ectopic endometrium samples detected using RT-qPCR (CON, *n* = 12; EU and EC, *n* = 16) and IHC (*n* = 10). **D** The mechanism of ferroptosis in endometriotic stromal cells. The iron-overload in cyst fluid triggered ferroptosis in cells of the inner surface of endometrioma and induced cytokines like VEGFA and IL8 secretion. The paracrine cytokines further promoted lesion angiogenesis to advance the development of endometriosis. Comparisons were made using one-way ANOVA (**B**, **C**). *P < 0.05, **P < 0.01, ***P < 0.001, ****P < 0.0001. ns not statistically significant, CON the control endometrium, EU eutopic endometrium from patients with endometriosis, EC ectopic lesions from patients with endometriosis, VEGFA vascular endothelial growth factor A, IL8 interleukin 8, IHC immunohistochemistry.

We further investigated the location and expression of VEGFA and IL8 in endometriosis biospecimens. Surprisingly, we found that VEGFA and IL8 were both highly expressed on the inner surface of ovarian cysts using IF, while no localised specific expression was observed in the control and eutopic endometria (Fig. 5A). Moreover, the expression of VEGFA and IL8 in lesions was significantly higher than that in the eutopic and control samples, as detected using RT-qPCR and IHC (Fig. 5B, C), indicating that VEGFA and IL8 expression might be closely related to the pathogenesis of endometriosis.

## Discussion

The term “ferroptosis”, defined as a distinct form of regulated cell death characterised by iron-catalysed lethal lipid peroxidation, was first coined by Dixon et al. [7]. Iron accumulation serves as an initial element in ferroptotic cell death [31]. Ovarian endometrioma is an ovarian cyst lined with endometrial tissue histologically and functionally resembling eutopic endometrium, which is generally considered to be filled with menstrual debris and antiquated blood [8]. Inside the cyst, the concentrations of free iron, ROS, and lipid peroxide are ten to hundreds of times higher than those in peripheral blood or other types of benign cysts, providing an iron overload and oxidative microenvironment for lesion growth [20, 32]. There is a point of view that the implanted ectopic endometrium can resist ferroptosis and survive in a microenvironment with iron overload due to dysregulated iron homoeostasis [33]. The latest meta-analysis of online datasets demonstrated that the ferroptosis pathway presents a downregulation trend among the ectopic, eutopic, and control endometria [34]. In contrast, according to previous studies, ectopic endometriotic lesions had significantly higher levels of ROS, hydrogen peroxide, and oxidative stress activity [35, 36]. Whether ferroptosis exists and has potential effects on the development of ectopic lesions has not yet been proven. Given that there is no gold standard to detect ferroptosis, we observed the ultrastructure of endometriotic cysts using TEM and found for the first time obvious mitochondrial morphological changes on its inner surface, consistent with the morphological features of ferroptosis. Moreover, we treated primary ESCs with cyst fluid in vitro and found a markedly elevated level of lipid ROS and shrunk mitochondria with increased membrane density, indicating that ESC ferroptosis was induced by the chocolate cyst fluid.

Surprisingly, we found that erastin-induced ESC ferroptosis could trigger the production of angiogenic, inflammatory, and growth cytokines, which may provide an original thinking to the positive effect of ferroptosis on the development of endometriosis. The small molecule erastin has been applied in many diseases as a ferroptosis trigger to explore the mechanism of this newly discovered non-apoptotic cell death in vitro [7, 31, 37]. Although a recent study showed that ectopic ESCs were more sensitive to erastin-induced ferroptosis than normal ESCs, suggesting that erastin may be a novel therapy for endometriosis [10], we show here that higher concentrations of erastin could trigger both eutopic and ectopic ESC ferroptosis and also subsequently induce the secretion of cytokines, such as VEGFA and IL8 (Supplemental Fig. 2A–C). VEGFA is generally regarded as a vital angiogenic factor, which attaches to the vascular endothelium to initiate cell proliferation and endothelial angiogenesis and increases vascular permeability [11]. Furthermore, IL8 signalling increases cell proliferation and survival to promote angiogenic responses in endothelial cells [38]. According to a study by Sun et al., IL6 and IL8 secretion was elevated in erastin-treated retinal pigment epithelial cells as senescence-associated factors [16]. Nevertheless, few other studies have investigated the potential role of ferroptosis in vascular formation. Our study is the first to demonstrate that ESCs in the process of ferroptosis, stimulate VEGFA and IL8 secretion, which may contribute to endometriotic lesion angiogenesis. Hence, ferroptosis might act as a double-edged sword in the progression of endometriosis. On the one hand, the agonist erastin might be a promising agent for triggering ferroptosis in lesions. On the other hand, some stromal cells undergoing ferroptosis secrete a series of cytokines to promote vascular system formation of surrounding tissues via paracrine actions, which may enhance benign cell proliferation and accelerate the progression of this disorder. Thus, we aimed to identify an inhibitor to attenuate cytokine secretion. Unfortunately, we have not investigated other cytokines, such as growth factors (ADM and AREG) and inflammatory factors (IL2), induced by ferroptosis. Further studies are also needed to explore more potential effects of ferroptosis on endometriosis.

According to our study, NAC could serve as an efficacious agent for suppressing ferroptosis-induced cytokine secretion. NAC, the acetylated precursor of l-cysteine, is generally considered as an anti-inflammatory or anti-oxidative agent. However, recent studies showed that NAC could also reverse lipid ROS levels and act as an inhibitor against ferroptosis [39, 40]. Its curative effect has been studied in a variety of diseases, such as Alzheimer’s disease, nephropathy, and heavy-metal toxicity [29, 41]. In endometriosis, animal experiments have already demonstrated its antioxidative functions in reducing the weight and size of ectopic lesions [19]. Moreover, in an observational cohort study, NAC was proposed as a promising treatment for endometriosis by decreasing the size and number of cysts, reducing dysmenorrhoea symptoms, and increasing chances of conception without side effects, but its mechanism has not been elucidated [42]. In this study, we incubated NAC with erastin-treated ESCs and surprisingly found that NAC rescued cell death morphologically and suppressed the secretion of VEGFA and IL8. Thus, it counteracted the stimulatory effect of ferroptosis on angiogenesis, providing a novel insight into mechanisms underlying the therapeutic effects of NAC in endometriosis.

Our study showed that the p38 MAPK/STAT6 signalling is one of the main downstream regulators of erastin-induced ferroptosis and that it mediates the secretion of VEGFA and IL8 in ESCs. A recent report revealed that ferroptosis inducers, such as RSL3 and erastin, induced the ASK1-p38 MAPK pathway activation downstream of lipid ROS in A549 cells [43]. JNK and p38, except for the ERK MAPK pathway, were also responsible for erastin-induced ferroptosis in an acute myeloid leukaemia cell line [44]. Furthermore, VEGFA expression was found to be regulated by activation of the p38 MAPK pathway, playing an important role in angiogenesis [45, 46]. We found increased phosphorylation of p38 MAPK in erastin-treated ESCs. The addition of a p38 inhibitor significantly repressed VEGFA and IL8 production. Other studies have shown that the coordinated modulation of STAT6 and p38 MAPK exhibited potential effects on the induction of IL4 and IL13 and that p38 MAPK could directly regulate the activity of the transactivation domain of STAT6 [47–49]. In erastin-induced ESCs, we also detected STAT6 activation, and transfection of *STAT6* siRNA attenuated the expression of VEGFA and IL8. Moreover, the luciferase reporter assay revealed a direct connection between STAT6 and both VEGFA and IL8. These data imply a critical link between the p38 MAPK/STAT6 pathway and ferroptosis-mediated VEGFA and IL8 expression in ESCs.

We also observed localised specific expression of VEGFA and IL8 in ectopic cysts and detected higher expression levels of VEGFA and IL8 in ectopic lesions, than in eutopic and control endometria in patients with endometriosis. Restricted by sample size, we failed to explore alterations in the expression levels and functions of VEGFA and IL8 during menstrual cycles. According to previous studies, the endometrial stromal cells present no differences in VEGF and IL8 expression throughout the menstrual phase [50, 51].

In conclusion, we detected ferroptosis caused by an iron overload on the inner surface of endometriotic cysts. Erastin-induced primary ESC ferroptosis stimulated VEGFA and IL8 secretion through the p38 MAPK/STAT6 pathway and promoted angiogenesis in vitro. Thus, ferroptosis may play a critical role in the progression of endometriosis by resulting in angiogenic effects via paracrine VEGFA and IL8 action on the adjacent lesions. NAC, which serves as a potential anti-ferroptosis agent, is expected to contribute significantly to the treatment of endometriosis.

## Methods

### Patients and sample collection

Thirty-six women of reproductive age who underwent laparoscopic and hysteroscopic procedures at the International Peace Maternity and Child Health Hospital (IPMCH), Shanghai Jiao Tong University School of Medicine, were recruited into this study. All enrolled women had regular menstrual cycles and did not receive hormonal therapy or use intrauterine contraception for at least 6 months prior to surgery. Patients with metabolic diseases, hypertension, inflammatory disease, autoimmune disorders, and cancer were excluded from the study. Twelve women with teratoma diagnosis who underwent combined hysteroscopy and laparoscopy according to their surgery options without macroscopic lesions in the uterine cavity served as the control group. Normal endometrial samples were obtained using an endometrial curette. Eutopic and ectopic endometrial tissues were collected from 24 patients with ovarian endometrioma using curette and laparoscopy, respectively. The demographic and baseline characteristics of patients in terms of age, body mass index, and parity had no significant difference between the control and endometriosis groups (Supplemental Table 1).

We dissected the samples using surgical scissors and tweezers and divided them into three groups. Group 1 samples were transported to the laboratory in phosphate-buffered saline (PBS) (Gibco, New York, USA) on ice for cell isolation. Group 2 samples were immediately fixed in 4% paraformaldehyde and then embedded in paraffin for immunohistochemical analysis. Group 3 samples were maintained in cryotubes and stored in liquid nitrogen for further RNA and protein extraction. The cyst fluid was aspirated by a 50 ml syringe and was then put in sterile 15 ml centrifugal tubes and stored at −80 °C until use.

The study protocol was approved by the ethics review committee of IPMCH and was conducted according to the principles of the Declaration of Helsinki. Written informed consent was obtained from all the participants.

### Isolation and culture of primary ESCs

Primary ESCs were isolated from the eutopic or ectopic endometrium of women with endometriosis. The tissues were cut into pieces and digested with type I collagenase (1 mg/ml, Gibco, New York, USA) for 0.5–1 h at 37 °C. After removing debris and epithelial cells using 100 and 40 μm cell strainers, respectively, ESCs were resuspended in DMEM/F12 containing 10% foetal bovine serum (FBS) (Gibco, New York, USA) and 1% penicillin–streptomycin (Gibco, New York, USA) and cultured in 5% CO_2_ at 37 °C. The culture medium was replaced after the stromal cells had attached, to remove blood cells and debris. After reaching 80–90% confluency in 2–3 days, cells were seeded into 12-well plates for in vitro experiments.

ESC purity was detected using immunofluorescence for vimentin and cytokeratin 7 as markers of stromal and epithelial cells, respectively. The number of vimentin-positive cells was greater than 95% (Supplemental Fig. 3).

### Cell cultures and treatment

The human embryonic kidney cell line HEK293T, human endometrial cancer cell line Ishikawa (ISK), human ovarian granulosa cell tumour cells (KGN), and human umbilical vein endothelial cells (HUVECs) were preserved in the Shanghai Key Laboratory of Embryo Original Diseases. HEK293T cells were cultured in DMEM High (Gibco, New York, USA) containing 10% FBS. ISK and KGN cells were grown in DMEM/F12 (Gibco, New York, USA) containing 10% FBS. HUVECs were incubated in an endothelial cell growth medium (PromoCell, Heidelberg, Germany) containing an endothelial cell growth supplement. All media were supplemented with 1% penicillin–streptomycin and cells were cultured in 5% CO_2_ at 37 °C. The medium was replaced every 2 days until 90% confluency was reached. All experiments were performed using cell lines from the fifth to tenth passage.

ESCs were treated with different erastin concentrations (10, 20, 30, 50, and 100 µM) for 12 h, and for different time periods (0, 3, 6, 9, and 12 h) with 30 µM erastin. Cell morphology was observed using an inverted microscope (Leica, Germany). To identify the specific effects of ferroptosis on ESCs, primary ESCs were treated with several ferroptosis inducers, such as erastin (30 µM), (1S,3R)-RSL3 (10 µM), tert-butyl hydroperoxide solution (TBHP, 20 µM), diluted cyst fluid (1:1 dilution with complete medium), and with inhibitor NAC (10 µM) for 12 h. Furthermore, HEK293T, ISK and KGN were treated with 30 µM erastin for 12 h. To explore the role of p38 MAPK in ferroptosis-induced VEGFA and IL8 induction, ESCs were treated with erastin in the absence or presence of the p38 inhibitor, SB203580 (10 µM) for 12 h. All reagents were purchased from Sigma-Aldrich (USA).

### Transmission electron microscopy (TEM)

Ectopic cyst walls were cut into 1 cm^3^ piece and fixed with 2.5% glutaraldehyde at 4 °C overnight immediately after they were obtained from the operating table. Primary ESCs were treated with or without the cyst fluid (1:1 diluted with complete medium) or dimethyl sulphoxide (DMSO) or 30 μM erastin for 12 h and then were washed thrice with PBS and fixed with 2.5% glutaraldehyde at 4 °C overnight. After washing in PBS twice for 10 min, the ectopic cyst wall pieces were fixed with 1% osmic acid at 4 °C for 2 h. Subsequently, the samples were dehydrated with an ethanol gradient and 100% acetone solution for 15 min and then embedded in epoxy resin. Ultrathin (70 nm) sections, obtained using an ultramicrotome, were stained with lead citrate and uranyl acetate for evaluation. Cell and mitochondrial morphology was captured using a transmission electron microscope (Hitachi H-7650, Japan).

### Analysis of lipid ROS accumulation

DMSO- or erastin (30 µM)- or cyst fluid-treated cells or cells from the ESC isolation procedures were incubated with C11-BODIPY (581/591) (Invitrogen, California, USA) for 30 min at 37 °C. Cells were subsequently resuspended in 1 ml of fresh PBS and strained through a 40 μm cell strainer for flow cytometry analysis. Lipid ROS levels were measured using an FFACScan (BD, New York, USA) through the FL1 channel (527 nm). Approximately 10,000 cells were analysed per sample. Data analysis was performed using the FlowJo version 10.0.

### RNA-seq, GO, and Kyoto encyclopaedia of genes and genomes (KEGG) analyses

Primary ectopic ESCs were seeded in 6 cm plates and treated with 30 µM erastin for 12 h. The cells from each group were collected using the RNAiso Plus reagent (Takara Bio, Tokyo, Japan) and sent to Annoroad Gene Technology (Beijing, China) for RNA-seq. The raw data were submitted to the GEO database (PRJNA783151). The raw RNA-seq data (FASTQ files) were filtered using the Perl script. Bowtie2 was used to build the genome index, and the RNA-seq data were then aligned to the reference genome using HISAT2. Read counts for each gene were counted using HTSeq, and fragments per kilobase million mapped reads were then calculated to estimate the gene expression level in each sample. DEGs were identified using the DESeq2 [52] package in R. The DEG threshold was set at *q* ≤ 0.05, and |log2_ratio | ≥1. DEG GO (http://geneontology.org/) and KEGG (http://www.kegg.jp/) enrichment were performed using the hypergeometric test, where the *p*-value was calculated and adjusted as a *q*-value. GO and KEGG terms with *q* < 0.05 were considered to be significantly enriched.

### Dual-luciferase reporter assay

The 2000 bp promoter sequence of *VEGFA* and *IL8* was searched on the UCSC website (http://genome.ucsc.edu/cgi-bin/hgGateway). Based on the obtained promoter sequence, we predicted the possible *signal transducer and activator of transcription 6 (STAT6)* transcription factor-binding region of the *VEGFA* and *IL8* promoter sequences. Surprisingly, we found a common predicted *STAT6* transcription factor-binding region in the *VEGFA* and *IL8* 2000 bp promoters (in the VEGFA mRNA 5′-UTR [GGGAAG, 1447–1452 nt] and IL8 mRNA 5′-UTR [AGGAAG, 1852–1857 nt)] regions). Then, we synthesised a target wild type (WT) sequence (GGGAAG/AGGAAG) and a mutant sequence (Mut) (GGGAAG/AGGAAG mutation) using site-directed mutagenesis. The synthesised WT or Mut *VEGFA* and *IL8* promoters were inserted into the KpnI and xhoI digestion sites of pGL4.22 vectors using the pEASY^®^-Basic Seamless Cloning and Assembly Kit (Transgen, Beijing, China). Recombinant plasmids were validated using sequencing. For the luciferase assay, HEK293T cells were seeded in 24-well plates until they reached 70% confluency and, subsequently, allocated into five groups: (1) transfected with GL-vector and Flag, (2) transfected with GL-VEGFA-WT and Flag-STAT6, (3) transfected with GL-VEGFA-Mut and Flag-STAT6, (4) transfected with GL-IL8-WT and Flag-STAT6, and (5) transfected with GL-IL8-Mut and Flag-STAT6. Cell lysates were harvested after 48 h of transfection and dual-luciferase reporter assays were performed using a Dual-Luciferase Reporter Assay System kit (Promega, Wisconsin, USA).

### siRNA transfection

Small interfering RNA (siRNA) targeting STAT6 was designed and synthesised by GenePharma (Shanghai, China), which also provided the negative control siRNA. The STAT6 siRNA sense and antisense strand sequences are presented in Supplemental Table 2. Primary ESCs were seeded into 12-well plates and cultured until they reached 60–80% confluency, followed by transfection with 20 pmol STAT6 siRNAs per well using the Lipofectamine® RNAiMAX reagent (Invitrogen, California, USA) in Opti-MEM (Gibco, New York, USA) according to the manufacturer’s instructions. After 48 h of transfection, the cells were treated with 30 µM erastin or DMSO for 12 h. To determine the transfection efficiency, quantitative real-time PCR (RT-qPCR) and western blot were performed 48 h after transfection.

### Total RNA extraction and RT-qPCR

Total RNA was extracted from tissues and cultured cells using the RNAiso Plus reagent (Takara Bio, Tokyo, Japan) in accordance with the manufacturer’s protocol. After RNA quantification using the Nanodrop 2000 (Thermo Fisher Scientific, USA), 1 µg of total RNA was reverse transcribed in a total volume of 20 μl using the PrimeScript^™^ RT reagent kit (Takara Bio, Tokyo, Japan). RT-qPCR was performed using TB Green Premix Ex Taq II (Takara Bio, Tokyo, Japan) and the QuantStudio 7 Flex Real‐Time PCR system (Applied Biosystems, USA). Primers details are mentioned in Supplemental Table 2. The 2^−ΔΔCt^ method was used to calculate the relative expression levels of target genes, which were normalised to those of actin beta mRNA levels.

### Protein extraction and western blot analysis

Cultured cells were lysed in radioimmunoprecipitation assay lysis buffer (Beyotime, Shanghai, China) supplemented with phenylmethylsulphonyl fluoride and protease inhibitor cocktail (Sigma-Aldrich, USA) on ice for 10 min and centrifuged at 12,000*g* for 10 min at 4 °C. Protein concentrations were quantified using a BCA assay kit (Beyotime, Shanghai, China). A total of 20 µg protein was separated using 10% or 12.5% sodium dodecyl sulphate polyacrylamide gel electrophoresis and blotted onto polyvinylidene fluoride membranes, which were then blocked with 5% non-fat milk diluted in Tris-buffered saline containing 0.05% Tween 20 for 1 h at room temperature. Blocking was followed by incubation with the primary antibodies, namely, anti-phospho-p38 MAPK (1:1000, Cell Signalling, 9211, Massachusetts, USA), anti-p38 MAPK (1:1000, Cell Signalling, 9212, Massachusetts, USA), anti-phospho-STAT6 (1:1000, Cell Signalling, 56554, Massachusetts, USA), anti-STAT6 (1:1000, Cell Signalling, 5397, Massachusetts, USA), anti-VEGFA (1:1000, Abcam, ab46154, Cambridge, UK), anti-IL8 (1:1000, Proteintech, 17038-1-AP, Chicago, USA), and anti-β-actin (1:5000, Proteintech, HRP-60008, Chicago, USA) at 4 °C overnight. The membranes were subsequently incubated with horseradish peroxidase-conjugated secondary antibodies (1:5000, Proteintech, SA00001-2, Chicago, USA) at room temperature for 1 h, and the signals were visualised using an enhanced chemiluminescence detection reagent (Sigma-Aldrich, USA).

### Enzyme-linked immunosorbent assay (ELISA)

The culture supernatant of ESCs was collected after treatment, centrifuged at 3000*g* for 5 min, and then stored at −80 °C until testing. VEGF and IL8 concentrations were measured using ELISA kits according to the manufacturer’s protocol (Neobioscience, Shenzhen, China).

### Immunofluorescence (IF)

ESCs were fixed with 4% paraformaldehyde at 4 °C for 10 min and then permeabilized with 5% Triton-100 at room temperature for 30 min. After deparaffinization, dehydration, rehydration and antigen retrieval, the paraffin sections were subjected to the same procedures. Samples were blocked with 5% bovine serum albumin (BSA) at room temperature for 1 h and subsequently incubated with primary antibodies against vimentin (1:100, Abcam, ab8978, Cambridge, UK), anti-cytokeratin 7 (1:100, Abcam, ab68459, Cambridge, UK), anti-VEGFA (1:100), and anti-IL8 (1:100) overnight at 4 °C. After washing with PBS, samples were incubated with Alexa Fluor 488- or 555-conjugated secondary antibodies (Invitrogen, California, A-21202/A-31572, USA) for 1 h at room temperature in the dark and stained with 4,6-diamidino-2-phenylindole (DAPI). Immunofluorescence was detected using a confocal microscope (Leica, Germany).

### Immunohistochemistry (IHC)

Fresh human specimens were fixed with 4% paraformaldehyde solution for 24 h, embedded in paraffin, and cut into sections (5 μm), which were then immersed in xylene and ethanol for deparaffinization and rehydration, respectively. Antigen retrieval was performed using Tris-EDTA (pH 9.0) (Biosharp, Anhui, China) in a microwave oven. The following procedures were performed using a staining kit (Absin, Shanghai, China). Briefly, the slides were incubated with 3% H_2_O_2_ to eliminate endogenous peroxidase activity and then blocked with 5% BSA followed first by incubation with primary antibodies against VEGFA (1:200) and anti-IL8 (1:200) overnight at 4 °C, and second by incubation with a secondary antibody. Staining was performed using DAB and haematoxylin. The slides were observed and imaged using a microscope (Leica, Germany). The VEGFA and IL8 protein expression levels were semi-quantitatively evaluated using the *H*-score system. The staining intensity was assessed using the scoring parameters: strong (3×), medium (2×), and weak (1×), and the *H*-score value ranging from 0 to 300 was calculated according to the formula [53, 54]: *H*-score = 1* (% cells 1×) + 2* (% cells 2×) + 3* (% cells 3×). The *H*-score was independently evaluated by two investigators at different times, and the average score was used.

### Matrigel tube formation assay

Primary ESCs were treated with erastin (30 µM) and/or NAC (10 µM) in serum-free DMEM/F12, and the supernatant was collected after 12 h of incubation. HUVECs were pre-treated with serum-free medium for 48 h before the Matrigel tube formation assay was performed. Subsequently, HUVECs were diluted with the supernatant or serum-free DMEM/F12 as a control and 50 µl of 10,000 cells/ well were added to a u-slide angiogenesis plate (Ibidi, Germany) precoated with BD Matrigel. Then, the slides were incubated at 37 °C for 6 h. Tube formation was imaged using an inverted microscope (Leica, Germany). The number of branches, an index of angiogenesis, was measured using ImageJ.

### Statistical analysis

All experiments were independently performed in at least triplicate. All statistical analyses were performed using GraphPad Prism version 8. The variances between the groups that are being statistically compared were similar. All data are presented as the mean ± SEM. The Student’s *t* test was used to analyse differences between paired data, whereas one-way ANOVA was used to analyse multiple comparisons. A *P* value of <0.05 was considered statistically significant.

## Supplementary information

Supplemental legends

Supplemental Table 1

Supplemental Table 2

Supplemental Figure 1

Supplemental Figure 2

Supplemental Figure 3

## Data Availability

The RNA-seq raw data have been deposited in the GEO database, which is available at https://www.ncbi.nlm.nih.gov/sra/PRJNA783151.
