# Supplementary material for: Endometrial stromal cell ferroptosis promotes angiogenesis in endometriosis
Source: Cell Death Discov. 2022 Jan 17;8:29. doi: 10.1038/s41420-022-00821-z (PMC8763888; doi:10.1038/s41420-022-00821-z)
Supplement: Supplementary file 1 — Supplemental legends [file 41420_2022_821_MOESM1_ESM.docx]

**Supplementary files**

**Supplemental Table 1. Baseline patient characteristics.**

^a^ Data are presented as the mean ± standard deviation. Differences were considered significant at p<0.05. ^b^ Statistical analysis was performed using the Student’s t-test or the Mann-Whitney U test.

**Supplemental Table 2. siRNAs and PCR primers used in this study**

**Supplemental Figure 1**

**Morphological changes of ESC under the treatment of erastin.** (A) ESCs were treated with erastin at different concentrations (10, 20, 30, 50 and 100 µM) for 12 h. (B) ESCs were treated with 30 µM erastin for different time periods (0, 3, 6, 9 and 12 h). Representative images are shown. ESCs, endometrial stromal cells.

**Supplemental Figure 2**

**ESCs from eutopic endometrium (EuESCs) and ectopic lesions (EcESCs) induced VEGFA and IL8 production under erastin treatment.** EuESCs and EcESCs were treated with 30 µM erastin in the absence or presence of NAC (10 µM) for 12 h. (A) The morphologic changes are shown. (B and C) mRNA and protein expression of VEGFA and IL8 in EcESCs detected using RT-qPCR and western blot. Three independent experiments were performed and comparisons were made using one-way ANOVA. **P*<0.05, ***P*<0.01, ****P*<0.001, *****P*<0.0001. ESCs, endometrial stromal cells; VEGFA, vascular endothelial growth factor A; IL8, interleukin 8, NAC, N-acetylcysteine.

**Supplemental Figure 3**

**The purity of ESCs**. (A) The purity of ESCs was confirmed using IF for the epithelial and stromal cell markers cytokeratin 7 (red) and vimentin (green), respectively. ESCs, endometrial stromal cells; IF, immunofluorescence.
