# Supplementary material for: Endometrial stromal cell ferroptosis promotes angiogenesis in endometriosis
Source: Cell Death Discov. 2022 Jan 17;8:29. doi: 10.1038/s41420-022-00821-z (PMC8763888; doi:10.1038/s41420-022-00821-z)
Supplement: Supplementary file 2 — Supplemental Table 1 [file 41420_2022_821_MOESM2_ESM.docx]

**Table 1. Baseline patient characteristics.**

| Patients’ characteristics | Control group  (n=12) | Endometriosis group (n=24) | P-value ^b^ |
| --- | --- | --- | --- |
| Age (years) ^a^ | 33.42±7.55 | 35.21±4.49 | 0.377 |
| BMI (kg/m^2^) ^a^ | 21.67±1.91 | 21.00±2.99 | 0.191 |
| Gravidity ^a^ | 1.42±1.38 | 1.08±0.88 | 0.639 |
| Parity ^a^ | 0.75±0.75 | 0.67±0.48 | 0.884 |
| Proliferation phase | 58.33% | 58.33% |  |

^a^ Data are presented as the mean ± standard deviation. Differences were considered significant at p<0.05. ^b^ Statistical analysis was performed using the Student’s t-test or the Mann-Whitney U test.
