# Supplementary material for: Endometrial stromal cell ferroptosis promotes angiogenesis in endometriosis
Source: Cell Death Discov. 2022 Jan 17;8:29. doi: 10.1038/s41420-022-00821-z (PMC8763888; doi:10.1038/s41420-022-00821-z)
Supplement: Supplementary file 3 — Supplemental Table 2 [file 41420_2022_821_MOESM3_ESM.docx]

**Table 2. siRNAs and PCR primers used in this study**

| Gene | Forward primer sequence | Reverse primer sequence |
| --- | --- | --- |
| STAT6 siRNA-1 | GCACCCUUGAGAGCAUAUATT | UAUAUGCUCUCAAGGGUGCTT |
| STAT6 siRNA-2 | GGCUGAUCAUUGGCUUCAUTT | AUGAAGCCAAUGAUCAGCCTT |
| β-actin | AGCGAGCATCCCCCAAAGTT | GGGCACGAAGGCTCATCATT |
| SAT1 | CCAATGTTCAAATGCGCAGC | TTTCTTCCCTTTGCGGACCA |
| PEBP1 | GCAATGACATCAGCAGTGGC | GGTGGTCTCCAGATCGGTTG |
| DPP4 | GGTTCTGCTGAACAAAGGCAAT | GCCTCCATTGCTTCACGTAG |
| GPX4 | TTCCCGTGTAACCAGTTCGG | GTGGAGAGACGGTGTCCAAA |
| DJ-1 | GCCTGGTGTGGGGCTTGTAA | GCTGGCATCAGGACAAATGAC |
| VEGFA | ACAAGATCCGCAGACGTGTA | TCACATCTGCAAGTACGTTCG |
| IL8 | ACTCCAAACCTTTCCACCCC | TTCTCAGCCCTCTTCAAAAACT |
| ANGPTL4 | TCTCTGGAGGCTGGTGGTTT | AGAGTCACCGTCTTTCGTGG |
| ADM | CTGCCCAGACCCTTATTCGG | ATCCGGACTGCTGTCTTCG |
| IL1A | CATTGGCGTTTGAGTCAGCA | CATGGAGTGGGCCATAGCTT |
| IL2 | TTCACAGTGTGTGGTCAACAT | TCTCTGCTCTAACACAGAGGGA |
| IL11 | GAGCGGACCTACTGTCCTAC | CTTCAGGGAAGAGCCACCTG |
| CLCF1 | AGAACTACGAGGCCTACAGC | AGGAGTCCAAGTGGGTTCAG |
| AREG | GTGTCCCAGAGACCGAGTTG | AGGCATTTCACTCACAGGGG |
| STAT6 | CAGTGACAGCGTTTCCTCAG | AGTGAGGTCCTGTTCAGTGG |
