# Supplementary figures and images for: Endometrial stromal cell ferroptosis promotes angiogenesis in endometriosis
Source: Cell Death Discov. 2022 Jan 17;8:29. doi: 10.1038/s41420-022-00821-z (PMC8763888; doi:10.1038/s41420-022-00821-z)

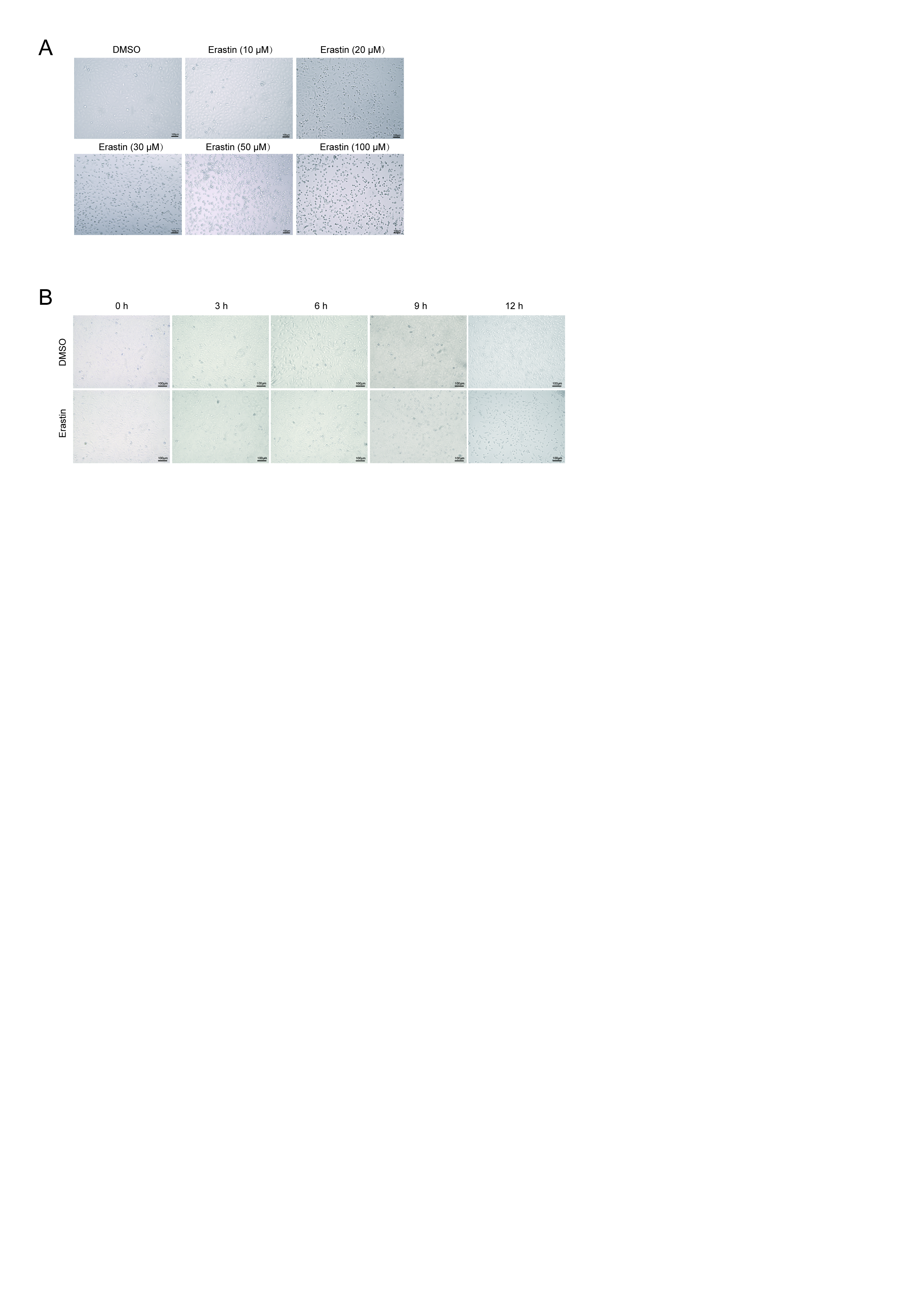

Supplement: Supplementary file 4 — Supplemental Figure 1 [file 41420_2022_821_MOESM4_ESM.tif]

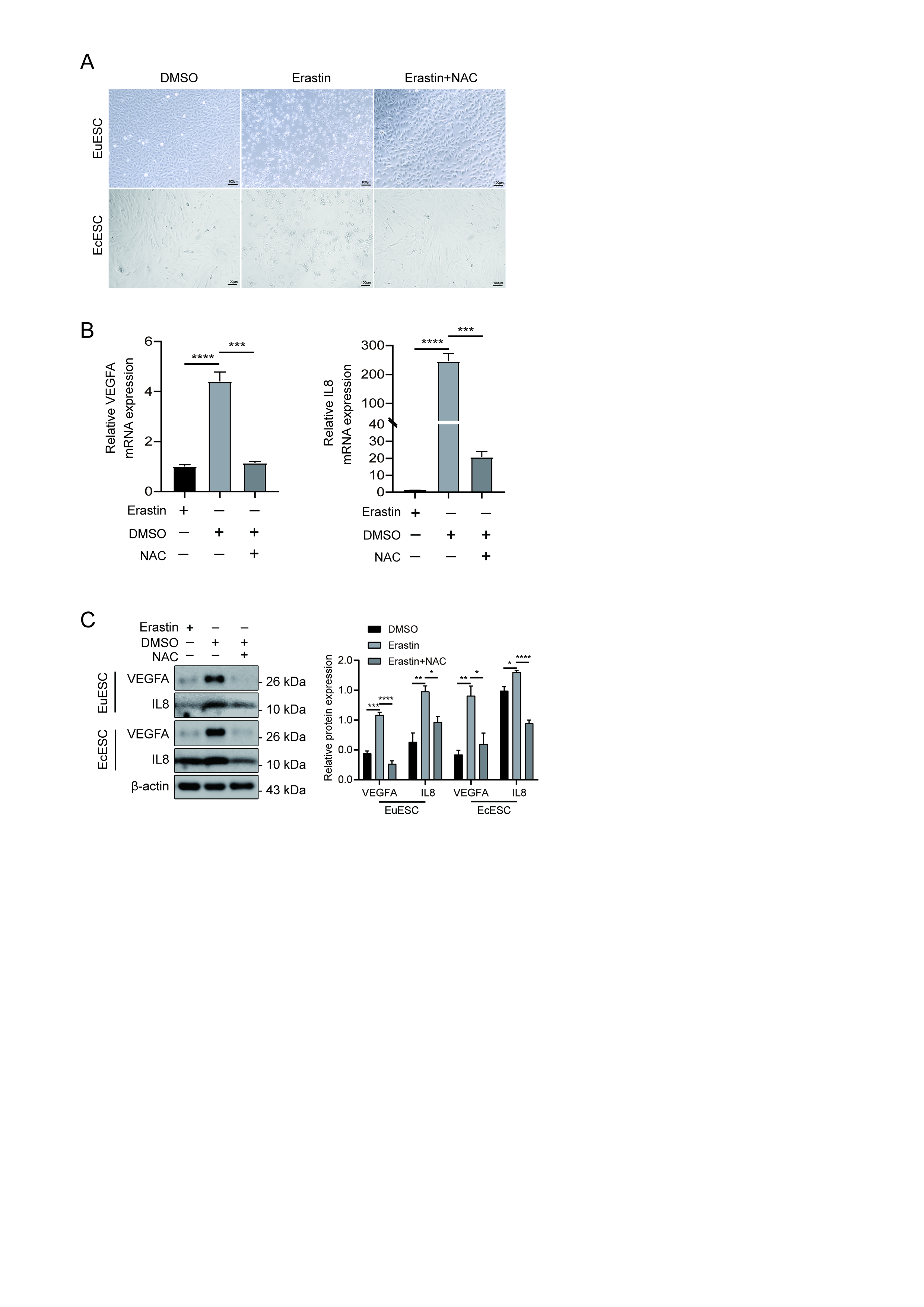

Supplement: Supplementary file 5 — Supplemental Figure 2 [file 41420_2022_821_MOESM5_ESM.tif]

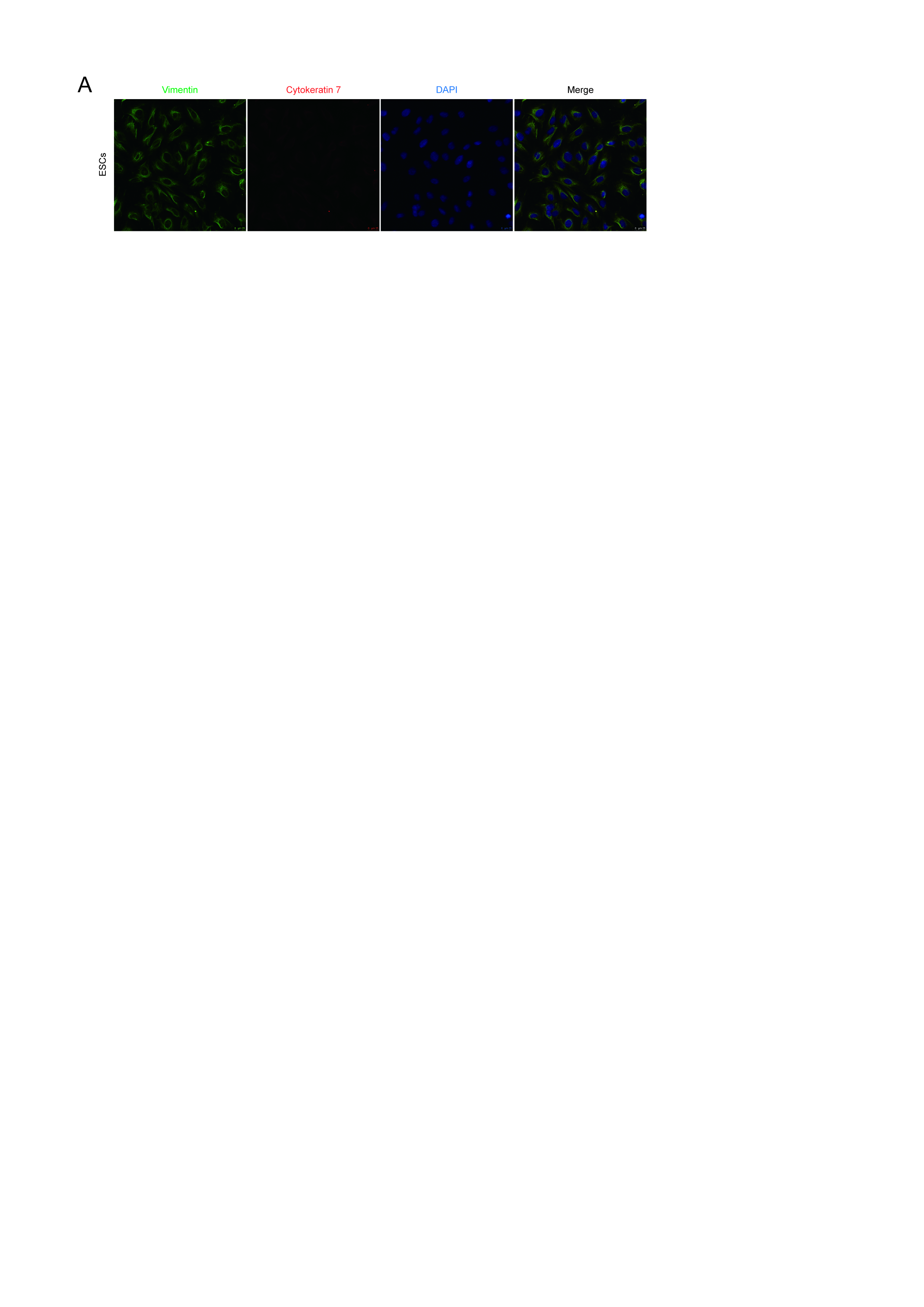

Supplement: Supplementary file 6 — Supplemental Figure 3 [file 41420_2022_821_MOESM6_ESM.tif]
